# Advancements and Challenges in Nanoscale Zero-Valent Iron-Activated Persulfate Technology for the Removal of Endocrine-Disrupting Chemicals

**DOI:** 10.3390/toxics12110814

**Published:** 2024-11-13

**Authors:** Dong Liang, Guoming Zeng, Xiaoling Lei, Da Sun

**Affiliations:** 1 Chongqing Academy of Science and Technology, Chongqing 401123, China; 2 School of Civil and Hydraulic Engineering, Chongqing University of Science and Technology, Chongqing 401331, China; 3 School of Civil Engineering, Chongqing Jiaotong University, Chongqing 400074, China; 4 National & Local Joint Engineering Research Center for Ecological Treatment Technology of Urban Water Pollution, College of Life and Environmental Science, Wenzhou University, Wenzhou 325035, China; 5 Zhejiang Provincial Key Laboratory for Water Environment and Marine Biological Resources Protection, College of Life and Environmental Science, Wenzhou University, Wenzhou 325035, China

**Keywords:** endocrine-disrupting chemicals, nZVI, advanced oxidation techniques, persulphate, toxicity

## Abstract

Endocrine-disrupting chemicals are a new class of pollutants that can affect hormonal metabolic processes in animals and humans. They can enter the aquatic environment through various pathways and gradually become enriched, thus posing a serious threat to the endocrine and physiological systems of both animals and humans. Nano zero-valent iron has promising applications in endocrine disruptor removal due to its excellent reducing properties and high specific surface area. However, given the dispersed focus and fragmented results of current studies, a comprehensive review is still lacking. In this paper, it was analyzed that the types of endocrine disruptors and their emission pathways reveal the sources of these compounds. Then, the main technologies currently used for endocrine disruptor treatment are introduced, covering physical, chemical, and biological treatment methods, with a special focus on persulfate oxidation among advanced oxidation technologies. Also, the paper summarizes the various activation methods of persulfate oxidation technology and proposes the nZVI-activated persulfate technology as the most promising means of treatment. In addition, this paper reviews the research progress of different modification methods of nZVI in activating persulfate for the removal of EDCs. Finally, the discussion includes recycling studies of nZVI/PS technology and emphasizes the urgency and importance of endocrine disruptor treatment. The review of this paper provides further scientific basis and technical support for nZVI/PS technology in the field of endocrine disruptor management.

## 1. Introduction

Endocrine-disrupting chemicals (EDCs) are environmental contaminants that interfere with the normal functioning of the endocrine system by mimicking or inhibiting hormone activities in organisms [1,2]. Often referred to as environmental hormones or endocrine disruptors, these compounds can bind to hormone receptors and disrupt physiological processes such as reproduction, development, and metabolism [3,4]. Although certain EDCs are typically present in low environmental concentrations, ranging from ng/L to μg/L, they possess bioaccumulation potential, particularly legacy pollutants such as dioxins and polychlorinated biphenyls (PCBs), which can pose significant adverse effects on wildlife and humans [5,6]. Additionally, other EDCs, such as plasticizers (e.g., phenols and phthalates), which are not bioaccumulative but are rapidly metabolized, also raise substantial environmental concerns [7]. The impact of EDCs is profound, as they can mimic natural hormones, trigger inappropriate cellular responses, or block hormone receptors, inhibiting normal signaling pathways [8,9].

**Figure 1:**
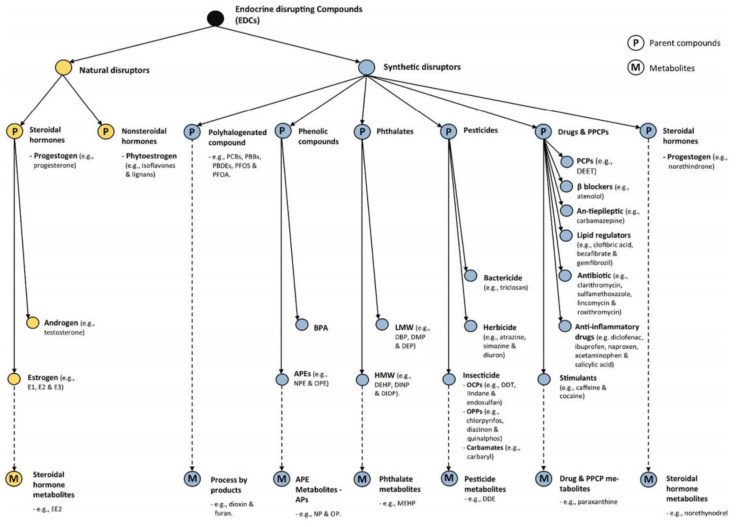
Hierarchy of EDCs [16] (copyright © 2017 Elsevier).

EDCs contain a wide variety of species, with up to 36,000 compounds currently suspected of interacting with the endocrine system [10,11]. As shown in Figure 1, These compounds can be broadly categorized into two groups: natural hormones and synthetic chemicals [12,13]. Natural hormones include natural estrogens (estrone (E1), 17β-estradiol (E2β), and estriol (E3)), natural androgens (testosterone, dihydrotestosterone, and androstenedione), and phytohormones (isoflavonoids and coumarins). These substances predominantly originate from human and animal excreta, especially from intensive livestock farming. On the other hand, synthetic chemicals encompass a wide range of industrial and household products. These include synthetic hormones (ethinylestradiol, norethindrone, and hexestrol), phenolic compounds (bisphenol A (BPA), nonylphenol, and alkylphenols), phthalates (dibutyl phthalate, bis (2-ethylhexyl) phthalate, and benzyl butyl phthalate), halogenated and aromatic substances (polycyclic aromatic hydrocarbons (PAHs), PCBs, dioxins, and furans), chemicals used in personal care products (parabens, musk fragrances, and anisole derivatives), and pesticide substances (DDT, lindane, and glyphosate). To address the continuous release and accumulation of EDCs in the environment, researchers have attempted various treatment methods, including physical, chemical, and biological approaches, to limit their environmental concentrations [14,15]. However, these methods generally exhibit low efficiency, resulting in the widespread persistence of EDCs in natural water bodies. Consequently, the development of more effective remediation technologies has become an urgent priority.

In recent years, the rapid advancement of nanotechnology has led to the exploration of nanoscale materials for environmental remediation [17,18]. Nano zero-valent iron (nZVI), in particular, has attracted significant attention due to its high surface area, strong reductive potential, and capacity to activate persulfate (PS), an oxidant with high efficacy in degrading organic pollutants [19]. The nZVI-activated persulfate system has shown considerable promise in the removal of EDCs from contaminated environments. For example, Kang et al. [20] demonstrated that nZVI, when combined with various oxidants (persulfate, peroxymonosulfate, and hydrogen peroxide), achieved superior removal efficiencies for pollutants like 1,4-dioxane (1,4-D) and arsenic (III), with the nZVI/PS system outperforming other combinations. Similarly, Barka et al. [21] reported that an nZVI/PS system could effectively remove BPA in wastewater, achieving removal rates exceeding 70% under optimized conditions. Guo et al. [22] used coconut shell biochar loaded with nZVI to activate PS for the removal of pyrene in PAHs. The results showed that 10 mg/L of PAHs could be completely removed within 60 min. While nZVI has been extensively studied, there is a notable gap in the literature concerning the comprehensive evaluation of modified nZVI and advanced oxidation techniques tailored specifically for EDCs removal.

This review aims to bridge this gap by providing a detailed examination of the sources and environmental prevalence of EDCs. It also evaluates the effectiveness of various nZVI modification strategies and advanced oxidation processes in enhancing EDC degradation. The review concludes with a discussion of the current challenges and future directions for the application of nZVI-activated persulfate technology in environmental remediation.

## 2. Sources and Contamination of EDCs

**Figure 2:**
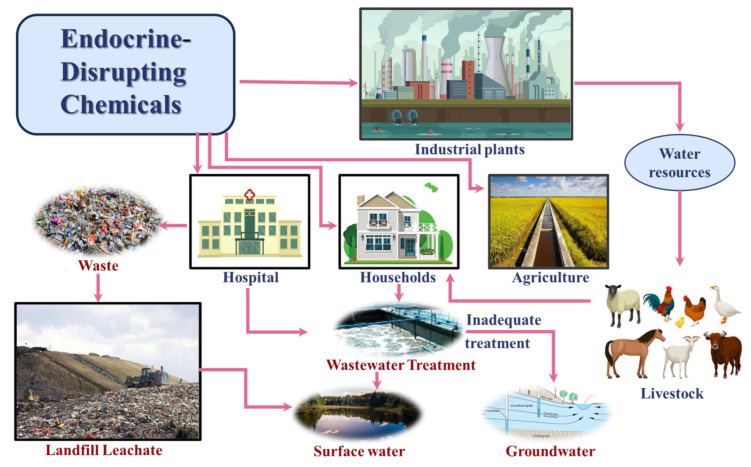
Releases and major sources of EDCs in water bodies.

EDCs were defined by the United States Environmental Protection Agency as “exogenous substances that interfere with the synthesis, secretion, transport, metabolism, binding, or elimination of naturally occurring hormones in the body, thereby affecting homeostasis, reproduction, and development. [23]”Later, the Endocrine Society redefined this definition, describing EDCs as “an exogenous chemical substance or chemical mixture that interferes with any aspect of hormone action [24].” Furthermore, with the publication of an increasing number of research findings regarding the hazards posed by environmental hormones to human health, the types of endocrine disruptors and their impacts and mechanisms of action have gradually garnered significant attention from the academic community and governments worldwide. For instance, the European Union mandates that the concentration of phenolic substances in detergents and pesticides must not exceed 0.1%. Japan ceased the use of PCBs and legislated that all entities in possession of PCBs must have properly disposed of them by 17 July 2016. Additionally, the Stockholm Convention on Persistent Organic Pollutants, signed internationally, initially identified 12 persistent organic pollutants that are prohibited for use. In April 2008, Canada declared BPA to be a harmful substance, leading some manufacturers to stop producing plastic bottles and baby products containing BPA. These regulations underscore the prevalence and complexity of endocrine disruptors, which can be found in various environmental matrices, including air, water, and soil (Figure 2).

EDCs enter the environment through numerous sources. Some originate from excreta, such as feces and urine, produced after the ingestion of drugs and their metabolites by humans, livestock, or aquatic organisms [25,26]. Others are released from industrial activities, agricultural runoff, sewage irrigation, medical wastewater, and landfill leachate [27,28]. These substances ultimately contaminate surface water and groundwater, posing a significant threat to environmental and public health. Additionally, through multimedia transfer, these contaminants can enter the food web, particularly bioaccumulative compounds, which pose further risks to human health as they accumulate in the tissues of organisms.

**Figure 3:**
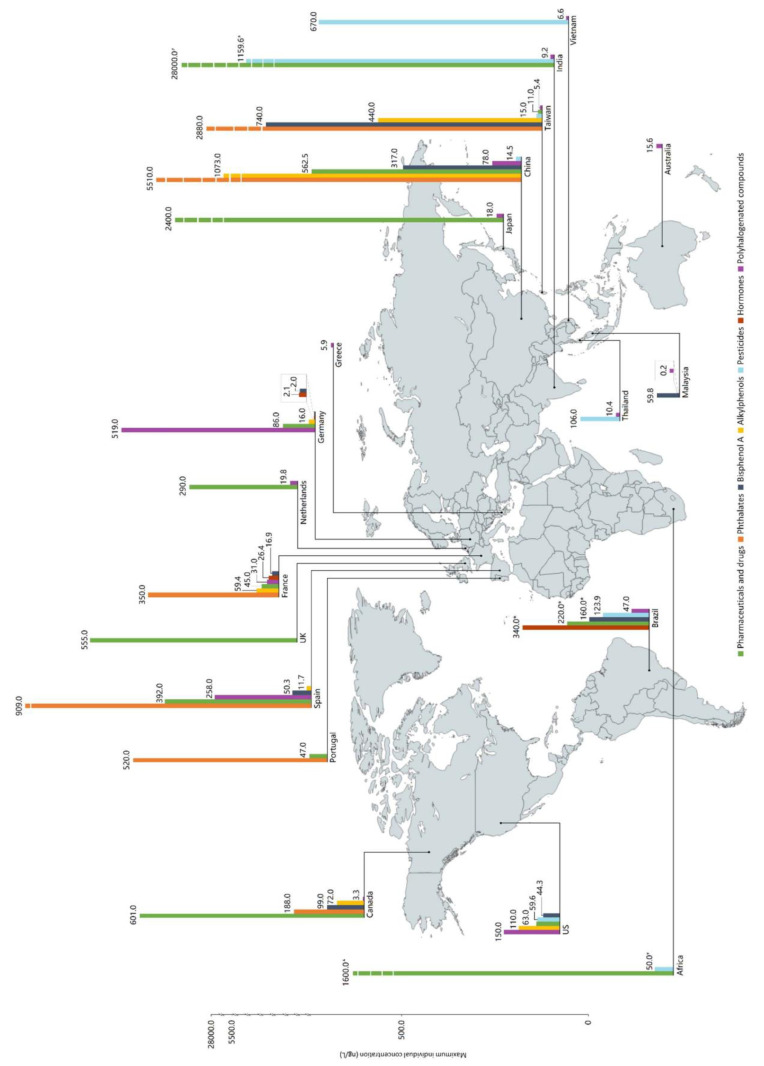
Global detection of EDCs in drinking water [16] (copyright © 2017 Elsevier).

The persistence of EDCs in the environment, particularly in aquatic ecosystems, is alarming because these compounds can exert adverse effects even at very low concentrations, often in the nanogram to microgram per liter range [29]. For instance, Lei et al. [30] examined estrogen levels in rivers within the Beijing–Tianjin–Hebei urban agglomeration in China, finding estrogen concentrations ranging from 23 to 255 ng/L. Similarly, studies on the Tamiraparani River in India revealed triclosan concentrations of 944 ng/L, which is significantly higher than levels found in other water systems [31]. At two monitoring stations in the river, triclosan levels ranged from 3800 to 5160 ng/L, far exceeding the acceptable concentrations. Additionally, EDCs have also been detected in drinking water systems worldwide [32]. Wee et al. compiled studies that show the presence of EDCs in drinking water across various regions globally (Figure 3) [16].

The above studies show that EDCs are indeed widespread in surface water. However, municipal wastewater and aquaculture wastewater are the most affected by EDCs [33,34]. Tang et al. [35] investigated the concentration of estrogens in municipal wastewater treatment plants and found 11 natural estrogens in the influent, with concentrations ranging from 7.9 to 62.9 ng/L. The five most concentrated natural estrogens in the influent were E1, E3, 16α-OHE1, 16ketoE2, and 2OHE1, with concentrations of 62.9, 62.6, 46.9, 32.7, and 28.8 ng/L, respectively. High estrogen levels in municipal wastewater influents are common due to daily discharges of estrogenic substances by humans. However, estrogens are still detectable in treated effluent from sewage treatment plants. Nasu et al. [36] conducted a study of 27 wastewater treatment plants and found that 15 EDCs exceeded the minimum detection limit in influent water, while 6 EDCs exceeded the minimum detection limit in effluent water. Among these, 17β-estradiol and nonylphenol polyethoxylates were detected in all influent samples. Similarly, a study by Petrie et al. [37] reported BPA concentrations as high as 100 μg/L in influent water from five sewage treatment works in the southwest of the UK. Despite removal rates exceeding 90% during wastewater treatment, effluent concentrations of BPA remained high, ranging from 62 to 892 ng/L. This is concerning as effluent is often chlorinated before discharge, potentially leading to the formation of chlorinated bisphenol products, which have been reported to exhibit significantly higher estrogenic activity than bisphenol itself [38]. Yost et al. [39] reported estrogenic activity in an anaerobic pond of a pig wastewater treatment plant, with levels reaching 5477 ng/L in the water column (expressed as E2β equivalents) and an alarming 1,302,795 ng/kg in the sludge. Estrogenic activity of chlorinated bisphenols was also detected in the water column of a pig wastewater treatment plant, reaching levels of 1477 ng/kg. Even surface runoff around agricultural fields treated with cow manure showed detectable levels of estrogen [40].

## 3. Current Status of Treatment Techniques for EDCs

### 3.1. Common Removal Techniques for EDCs

Most EDCs possess excellent lipid solubility, hydrophobicity, and chemical stability, allowing them to effectively bind to soil and sediment to form stable structures. Additionally, these compounds are challenging to degrade or eliminate due to their long half-lives and the low doses required for their activity. Advanced oxidation techniques are currently the leading methods for treating EDCs [41], but other approaches, such as adsorption, membrane filtration, biodegradation (bacteria, algae, and fungi), and coagulation, have demonstrated unique advantages [42,43,44].

Adsorption technologies are particularly effective in removing EDCs due to the large specific surface area and porosity of adsorbent materials, which facilitate effective interactions with these compounds. Adsorbent materials can achieve a high adsorption capacity for BPA, reaching up to 1113 mg/g [45]. However, recycling and desorption of adsorbent materials can be difficult, leading to high costs and limiting their practical applications. Membrane filtration relies on the separation principles of a semi-permeable membrane that traps organic pollutants, offering high effluent quality and low concentrations of organic matter. However, once the membrane becomes saturated, contaminants may desorb and re-enter the water. Biodegradation typically involves the breakdown of EDCs through microbial growth and metabolic processes, which can be categorized into anaerobic microbial degradation [46], aerobic microbial degradation [47], and sequential anaerobic–aerobic microbial degradation [48]. This approach is promising due to its low cost, environmental friendliness, and ability to maximize the mineralization of pollutants. Nonetheless, the slow degradation rate and lengthy remediation cycle necessitate enhancements to accelerate the microbial degradation process. Coagulation removes pollutants from water bodies through solid–liquid separation by adding coagulants that alter the physicochemical properties of suspended particulate matter and colloidal particles, causing them to destabilize and aggregate into larger particles [49].

The mechanisms of action and advantages and disadvantages of commonly used EDC removal techniques are presented in Table 1.

Advanced oxidation technologies (AOTs) degrade EDCs in aqueous phases or the environment by generating free radicals under specific conditions. These radicals, with their strong oxidative properties, can degrade EDCs into low or non-toxic, readily biodegradable intermediates or mineralize them directly into inorganic substances. AOTs can generate strongly oxidizing radicals in several ways (see Table 2), with the primary methods being photocatalytic oxidation, Fenton oxidation, electrochemical oxidation, and persulfate oxidation.

In photocatalytic methods, light intensity and wavelength are critical factors in determining the effectiveness of EDC degradation. Polybrominated diphenyl ethers (PBDEs) can be photolytically converted to Polybrominated dibenzofurans (PBDFs) in the wavelength range of 280 nm to 400 nm [50], and sunlight can also induce PBDE debromination. Bezares-Cruz et al. [51] found that nonabromodiphenyl ethers could convert to tribromodiphenyl ethers within 34 h under sunlight. However, this method faces challenges such as low light energy utilization, difficulties in recycling catalysts, and the tendency for photogenerated electron–hole pairs to recombine and become inactive. The Fenton oxidation technique involves the reaction of H_2_O_2_ with Fe^2+^ to form hydroxyl radicals under acidic conditions, with the H_2_O_2_ and Fe^2+^ mixture known as Fenton’s reagent. Zhang et al. [52] used nZVI as a catalyst to activate H_2_O_2_ (at a concentration of 20 mmol/L) under acidic conditions (pH = 3–4). With a catalyst dose of 100 mg/L, 95% of norfloxacin (NOR) was removed after 40 min, with a mineralization rate of approximately 50%. However, the catalyst is expensive and difficult to separate and reuse. The electrochemical method separates EDCs from contaminated media by utilizing their charge-directed movement in a low-voltage electric field generated by two electrodes inserted into the contaminated environment. Electrochemical cleavage experiments on PBDEs in tetrahydrofuran (THF) solution by Konstantinov et al. [53] showed lower levels of brominated diphenyl ethers at the beginning of electrolysis. Electrochemical debromination also preferentially occurs at the inter- and para-sites of the ether bond. However, this method requires an applied current and involves high energy consumption.

Among these methods, persulfate oxidation stands out for its high oxidizing capacity, ability to effectively remove a wide range of toxic and difficult-to-degrade organics, low operating costs, simplicity, and rapid degradation. As a result, it has gained significant attention and is currently the most widely used advanced oxidation technology [54].

### 3.2. Persulphate Oxidation Technology

Persulfates, such as peroxodisulfates (PS, S_2_O_8_^2−^) and permonosulfates (PMS, HSO_5_⁻), are environmentally friendly oxidants used in AOPs for the degradation of various organic pollutants, including EDCs. These compounds contain peroxy groups (-O-O-), similar to hydrogen peroxide (H_2_O_2_). Under ambient conditions, both PMS and PS are stable and soluble in water but require additional energy or activation by transition metals to break the -O-O- bonds and generate strongly oxidizing SO_4_^−^ with high oxidation potential, which can effectively degrade EDCs through a series of oxidative reactions (Equations (1)–(5)).

S_2_O_8_^2−^ → 2 SO_4_^−^ (1)

HSO_5_^−^ → SO_4_^−^ + OH^−^ (2)

SO_4_^−^ + H_2_O → HSO_4_^−^ + OH (3)

SO_4_^−^ + OH^−^ → SO_4_^2−^ + OH (4)

2·OH + 2 H^+^ + 2e^−^ → 2H_2_O (5)

Advanced oxidation techniques based on SO_4_^−^ possess higher oxidation potentials. Sulfate radicals effectively target organic compounds through electron transfer, especially in unsaturated π-bonds or aromatic functional groups. Additionally, the half-life of SO_4_^−^ (3040 μs) is generally longer than that of -OH (<1 μs), providing sufficient time to react with pollutants in water [55]. Persulfate-based advanced oxidation technologies are less affected by pH and have a broader operational range. Unlike -OH, which non-selectively attacks organic pollutants via electrophilic addition or dehydrogenation, SO_4_^−^ is more selective and can directly target specific organic functional groups [56]. However, because persulfates are not easily activated at room temperature, selecting an appropriate activation method is crucial for effective EDC degradation.

#### 3.2.1. Ultraviolet Activation

Ultraviolet (UV) activation involves the photolysis of PS when exposed to UV light, which breaks the O-O bond to generate SO_4_^−^. This radical initiates the degradation of pollutants, leading to their mineralization into smaller molecules (Equation (6)):

S_2_O_8_^2−^ + UV → 2 SO_4_^−^ (6)

UV-activated persulfates are particularly effective in the degradation of a wide range of organic pollutants. Fan et al. [57] demonstrated that under alkaline conditions, UV-activated PS can convert perfluoroalkyl and polyfluoroalkyl substances’ (PFASs) precursors into detectable perfluoroalkyl acids (PFAAs) within one hour. Yan et al. [58] investigated β-N-methylamino-L-alanine (BMAA) degradation in a UV/PS system, optimizing experimental parameters to achieve a pseudo-first-order rate constant of 1.039 min⁻^1^ under slightly alkaline conditions. Three potential transformation products of BMAA were identified: 2,3-diaminopropionic acid, L-alanine, and 2-hydroxy-3-methylaminopropionic acid. Additionally, Khan et al. [59] removed 93.2% of lindane using UV-activated persulfate under a fluence of 720 mJ/cm^2^ but noted that humic acid and other co-existing anions inhibited an oxidative degradation reaction. Although UV activation of persulfate is relatively straightforward, UV light can be partially absorbed or blocked by other substances, limiting its suitability for treating EDCs in soil or sediment.

#### 3.2.2. Thermal Activation

Thermal activation is another widely used method to enhance the reactivity of persulfates. As the temperature increases, the activation energy required to break the O-O bond in persulfate decreases, resulting in the decomposition of one PS molecule into two sulfate radicals (Equation (7)). Elevated temperatures also promote the formation of HO⁻, which further reacts with S_2_O_8_^2−^ to generate SO_4_^−^.

S_2_O_8_^2−^ + heat → 2 SO_4_^−^ (7)

Wang et al. [60] used thermally activated PS for degrading diethyl phthalate (DEP) and dibutyl phthalate (DBP), finding that the reaction rate increased with temperature and that lower pH facilitated pollutant degradation. Similarly, the degradation rate of thiamethoxam (TMX) was positively correlated with temperature, achieving maximum degradation at pH 6 with an activation energy of 108.7 kJ/mol [61]. However, a higher temperature does not always enhance degradation rates, especially for chlorinated alkanes with strong oxidation resistance. Thermal activation does not introduce new substances and involves a straightforward mechanism, making it suitable for in situ chemical oxidation (ISCO) [62]. However, thermal activation requires precise control of temperature conditions to prevent excessive energy consumption and to avoid the formation of unwanted byproducts.

#### 3.2.3. Alkaline Activation

Alkaline activation of PS involves adding alkaline reagents, like sodium hydroxide or calcium hydroxide, to create an alkaline environment conducive to PS activation. The degradation mechanism of organic pollutants by alkaline-activated PS varies with pH. Under acidic or neutral conditions, SO_4_^−^ plays a dominant role, while under alkaline conditions, SO_4_^−^ generates -OH, which degrades pollutants through addition or hydrogen capture, triggering a free radical chain reaction. This chain reaction produces superoxide radicals (O_2_^−^), enhancing degradation (Equations (8)–(10)).

S_2_O_8_^2−^ + 2H_2_O +OH^−^ → 2 SO_4_^−^·+ SO_4_^−^ + O_2_^−^ + 4H^+^ (8)

S_2_O_8_^2−^ + HO_2_^−^ + OH^−^ → SO_4_^2−^ + SO_4_^−^ + O_2_^−^ + H^+^ (9)

SO_4_^−^ + OH^−^ → SO_4_^2−^ + HO∙ (10)

Garcia-Cervilla et al. [63] explored combining a nonionic surfactant with sodium dodecyl sulfate (SDS) for site remediation in an alkali-activated PS system. Nonionic surfactants enhanced PS consumption, achieving 60% chloride conversion with E-Mulse3 (E3) versus less than 20% with SDS. NaOH-activated PS effectively treated chlorinated organic compound (COC)-contaminated soil, with conversion rates of 96% (for particles 2 mm > dp > 0.25 mm) and 70% (for particles dp < 0.25 mm) within 21 days [64]. The system also degraded benzo(a)pyrene, benzo(a)anthracene, and total petroleum hydrocarbons (TPHs) by 60–90%, with degradation efficiency increasing with water migration and depth [65]. Alkaline activation is easy to control, and the radicals can be identified and analyzed, making it suitable for alkaline polluted environments. However, high pH can corrode equipment and damage certain soil components, resulting in high reprocessing costs, which limits its practical use.

#### 3.2.4. Transition Metal Activation

Transition metal activation involves using transition metal ions (Fe^2+^, Cu^2+^, Mn^2+^, etc.) to provide electrons that catalyze the cleavage of the O-O bond to form SO_4_^−^ (Equation (11)):

M^n+^ + S_2_O_8_^2−^ → M^(n + 1)+^ + SO_4_^−^·+ SO_4_2^−^ (11)

Zhang et al. [66] synthesized an effective Co_3_O_4_/ZnO catalyst with an enriched Co_3_O_4_-ZnO interface that facilitated electron redistribution, accelerating electron transfer between the catalyst and persulfate (PMS). Co_3_O_4_/ZnO-3 showed higher catalytic activity for PMS than Co_3_O_4_ alone, achieving 100% phenol removal within 10 min. Wang et al. [67] used Fe^2+^, Co^2+^, Cu^2+^, and Mn^2+^ to activate persulfate for the decomposition of anaerobically digested sludge (ADS). They found that capillary suction time (CST) was lowest when PS was activated by Fe^2+^ and Cu^2+^, while Co^2+^ significantly reduced total dissolved oxygen (TDO) in extracellular polymers (EPSs) to 496.6 mg/L. Among these, Fe^2+^ is widely used in persulfate activation systems due to its simple preparation and low cost. Guo et al. [68] investigated the effectiveness of Fe^2+^/persulfate in degrading pyrene in soil through intermittent and column experiments, achieving approximately 93.2% degradation at a persulfate concentration of 65 mM and an Fe^2+^/persulfate molar ratio of 0.25. Dielectric barrier discharge (DBD) plasma significantly enhanced the degradation efficiency of Fe^2+^/PS against nitrophenol (PNP), with increased -OH and SO_4_^−^ production being key factors for improved degradation [69]. Transition metal activation, particularly Fe^2+^, is widely used for EDC removal due to its speed, efficiency, and wide applicability without needing additional heat or light sources. However, the instability and rapid depletion of Fe^2+^ limit further research and application, as Fe^2+^ tends to react with SO_4_^−^, reducing the degradation efficiency of EDCs.

#### 3.2.5. Nano Zero-Valent Iron Activation

Fe^0^ can serve as both a direct activator for PS, generating SO_4_^−^, and as a reducing agent for Fe^3+^ under specific conditions. Fe^0^ can also release Fe^2+^ through other reactions to mitigate the issue of excessive Fe^2+^ consumption, as illustrated in reaction Equations (12)–(15):

Fe^0^ + S_2_O_8_^2−^ → Fe^2+^ + 2 SO_4_^−^ + SO_4_^2−^ (12)

Fe^0^ + 2Fe^3+^ → 3Fe^2+^ (13)

Fe^0^ + H_2_O + 1/2 O_2_ → Fe^2+^ + 2OH^−^ (14)

Fe^0^ + 2H_2_O → Fe^2+^ + 2OH^−^ + H_2_↑ (15)

**Figure 4:**
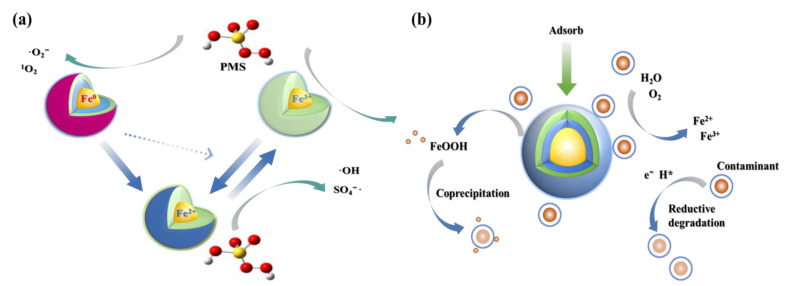
(**a**) The mechanisms of nZVI-catalyzed peroxynitrite and (**b**) nZVI adsorption and reduction of emerging pollutants. H*: atomic hydrogen.

As shown in Figure 4a, Fe^0^ functions as a heterogeneous catalyst, gradually releasing Fe^2+^ and controlling the reaction rate to ensure the continuous and efficient degradation of pollutants. Peluffo et al. [70] compared the effectiveness of PS activated by nZVI, Fe^2+^, and Fe^3+^ in degrading PAHs in soil. The nZVI/PS system demonstrated excellent degradation performance for phenanthrene (Phe) and benzo(a)pyrene (Pyr). Additionally, nZVI/PS was effective in degrading tetrabromobisphenol A (TBBPA) in soil, achieving up to 78.32% degradation at an nZVI concentration of 3 g/kg. Coexisting ions like Cu(II) enhanced the degradation effect, while Zn(II) inhibited it. Zhang et al. [71] explored the degradation of NOR using the nZVI/PS system under varying conditions of pH, temperature, nZVI dosage, and PS concentration. The optimal removal rate of 93.8% was achieved with 0.1 g/L nZVI and 12 mM PS, with hydroxyl radicals playing a dominant role. nZVI can effectively degrade not only synthetic compounds but also natural hormones, which are frequently detected in wastewater and surface water, posing potential threats to ecosystems and human health. Due to its strong reducing and adsorptive properties, nZVI can disrupt the chemical structure of natural hormones, significantly reducing their bioactivity. Ding et al. [72] used nZVI to activate peroxynitrite for the removal of E2, demonstrating that Fe⁰ and Fe^2^⁺ were the primary catalytic activators. PS is activated by electrons produced from the redox reactions between Fe^2+^ and Fe^3+^, generating free radicals. In conclusion, nZVI has emerged as a highly favored technology for environmental remediation in recent years, owing to its low cost, large specific surface area, sustained supply of Fe^2+^ for direct or indirect reactions with PS to degrade target pollutants, and the stability of the activated persulfate [73].

## 4. Progress of Nano Zero-Valent Iron-Activated Persulfate for Removal of EDCs

nZVI refers to iron particles with a size range of 1–100 nm, characterized by a large specific surface area and high reactivity [74,75]. In addition to the strong reducing capability of nZVI itself, the iron oxides formed upon its oxidation also exhibit excellent adsorption properties. As shown in Figure 4b, nZVI can directly reduce pollutants or convert them into less toxic products by using its strong reducing properties (*E*_0_ = −0.44 V), transforming them into low-valence, non-toxic, and insoluble states [76,77]. Moreover, as an efficient catalyst, nZVI can generate reactive oxygen species to oxidize pollutants. Additionally, nZVI’s adsorption properties enable it to immobilize EDCs in water, such as pentachlorophenol (PCP), PCBs, and TBBPA. nZVI is also considered environmentally benign due to its low toxicity. Liu et al. [78] demonstrated that adding 100 mg/kg of nZVI to PCP-contaminated soil could enhance rice yield and quality, ensure safe rice production, and promote the remediation of PCP-contaminated soils through rhizosphere microorganisms. Furthermore, nZVI can be easily separated and removed by simple magnetic separation, minimizing potential environmental risks [79]. In summary, nZVI is a versatile and environmentally friendly material with broad application prospects.

However, nZVI particles have limited stability, posing certain challenges for practical applications [80]. Firstly, nZVI is prone to oxidation by air, leading to the formation of a passivation layer that reduces its reactivity. Additionally, nZVI tends to agglomerate due to its small particle size and strong magnetic properties, resulting in a decreased specific surface area and reaction rate. Furthermore, nZVI may undergo spontaneous combustion under aerobic conditions, posing safety concerns during its handling and application.

**Figure 5:**
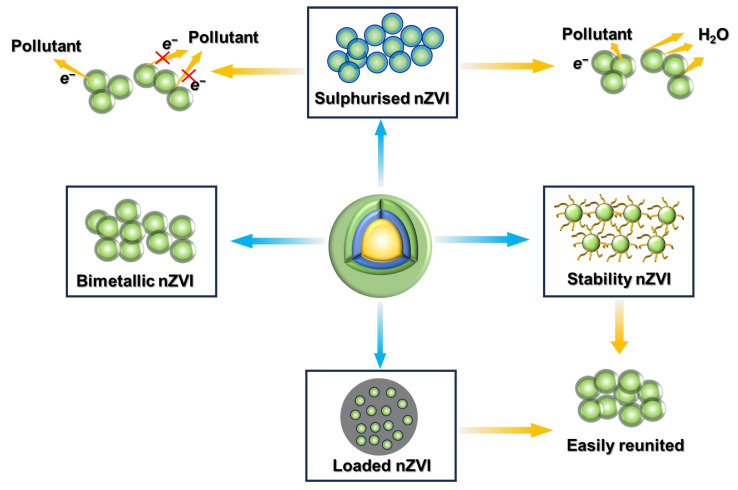
Application defects and common modifications of nZVI (Blue arrows indicate the modification technique and green arrows indicate the shortcomings of the technique).

To address these challenges, various modified strategies have been developed to enhance the stability and reactivity of nZVI. These modifications can be broadly categorized into four main approaches (Figure 5): (1) stabilized nZVI, where surfactants are added to increase reactivity and mobility by enhancing spatial resistance and electrostatic repulsion between nZVI particles; (2) supported nZVI, where nZVI is uniformly distributed on carriers such as biochar, kaolinite, and bentonite to prevent agglomeration and enhance reactivity; (3) bimetallic nZVI, where noble metals are doped into nZVI to improve its reactivity through catalytic effects on hydrogen (H₂); and (4) sulfur-modified nZVI, where elemental sulfur is doped to enhance the reactivity and selectivity of nZVI. These modifications not only improve the performance of nZVI in environmental remediation but also extend its applicability to a wider range of contaminants under various environmental conditions. Table 3 summarizes the removal efficiencies of different modified nZVI materials on endocrine disruptors.

### 4.1. Surface Modification nZVI

Surface modification methods involve introducing polymers or surfactants during the preparation of nZVI [93]. Due to weak van der Waals forces, high surface energy, and inherent paramagnetism, untreated nZVI tends to agglomerate, making it challenging to fully react with target pollutants, thus limiting its practical applications.

To address these issues, researchers have developed various surface-modified nZVI materials by adding chemically modified functional groups (both covalent and non-covalent) or stabilizers, such as soluble polymers or surfactants, to the surface of nZVI. These surface modifications alter the surface charge of nZVI, reduce electrostatic attraction and aggregation between particles, and thereby improve its dispersibility and mobility in aqueous media. For instance, Chen et al. [81] modified nZVI with polyvinylpyrrolidone (PVP), which reduced the particle size and increased the specific surface area, significantly enhancing the removal efficiency of tetracycline (TC). When a moderate amount of polymer (at concentrations of around 1.0 g/L or less) is added to the colloidal dispersion, most of the polymer adheres to the particle surface. This not only increases the repulsive forces between particles but also enhances electrostatic stability, thereby improving the mobility of nZVI. Additionally, different surfactants have been found to significantly enhance the reductive debromination of nZVI for PBDEs, as demonstrated by Liang et al. [94]. Among these, nonionic surfactants (Triton X-100) were the most effective in enhancing the debromination rate, followed by cationic surfactants (CPCs), while anionic surfactants (SBBDSs) had a relatively weaker effect.

### 4.2. Load-Modified nZVI

Load-modified nZVI involves incorporating carrier materials during synthesis so that nZVI is supported on solid substrates (biochar, kaolin, bentonite) to enhance its stability [95]. Qiu et al. [82] discovered that SiO₂-loaded nZVI, when activated with peroxynitrite and combined with sodium dodecyl sulfate (SDS) for soil washing, could degrade 75% of phenanthrene (PHE), 85% of fluoranthene (FLU), and 87% of pyridinol (PYR) within 30 min. Diao et al. [83] effectively degraded 2,4-dichlorophenol (2,4-DCP) using the B-nZVI/PS system, achieving a maximum degradation rate of 91% at slightly acidic pH. They also examined the negative impact of humic acid (HA) on 2,4-DCP degradation, identified dominant radical species through burst experiments, and analyzed the 2,4-DCP degradation pathway and Cd(II) immobilization mechanism. These studies indicate that the uniform distribution of nZVI on carriers like clay materials, biochar, and resin can effectively combine the adsorption properties of the carriers, improving the degradation efficiency of EDCs when combined with persulfate activation.

Biochar (BC), a material produced by the pyrolysis of organic matter at optimal temperatures (approximately 400–700 °C), is distinct from traditional charcoal due to its electron-rich surface and carbon–oxygen groups. Most biochar precursors are sourced from agricultural and forestry residues, fruit shells, and sludge, making them readily available [96]. Biochar has a high carbon content, a porous structure, a large specific surface area, and stable chemical and physical properties, making it widely studied for nZVI carrier applications. The calcination temperature of biochar significantly influences its carbon structure and hydrophilicity, while pyrolysis temperature affects its carbonization degree, carbon content, and water adsorption capacity. The feedstock used for biochar production also plays a crucial role in its performance. Diao et al. [86] found that the BC-nZVI/PMS system exhibited excellent atrazine degradation (96%, involving dealkylation, dechlorination, and hydroxylation) due to the involvement of SO_4_^2−^ reactive oxygen species, with OH and ^1^O_2_ also participating in the reaction. Li et al. [84] synthesized BC-nZVI using straw-derived biochar via liquid-phase reduction and demonstrated that 82.06% of BDE209 could be removed within 240 min with persulfate (PS) addition, with SO_4_^2−^ being the predominant reactive species in both acidic and neutral conditions. The BC-nZVI/PS system also effectively removed Cu^2+^ and BPA, with removal efficiencies exceeding 95%. A synergistic effect between Cu^2+^ and BPA removal was also observed [85].

### 4.3. Bimetallic-Modified nZVI

Bimetallic-modified nZVI involves doping metal layers (Ni, Cu, Pt) on the surface of nZVI to facilitate electron transfer from Fe^0^ to the nZVI surface. Compared to nZVI alone, bimetallic-modified zero-valent iron offers several advantages, such as higher reactivity and reduced deposition of corrosion products [97]. This modification effectively prevents the oxidative deactivation of nZVI, enhancing its degradation performance for target pollutants. Zhang et al. [87] synthesized Fe/Ni bimetallic nanomaterials for decabromodiphenyl ether (BDE209) removal, finding that adding Ni significantly improved reaction efficiency, achieving nearly complete removal within 180 min. Xu et al. [88] developed Fe/Cu bimetallic zero-valent iron catalysts activated with surfactants to degrade 2,4-dichlorophenol (DCP) in soil effectively, achieving a maximum degradation rate of 86%, the degradation efficiency of DCP was positively correlated with initial PS concentration. Researchers also explored the role of bimetallic Fe/Cu catalysts in PS activation, revealing a synergistic effect of Cu and Fe, with Cu atoms serving as the primary reaction sites for DCP decomposition while Fe atoms enhanced Cu’s activity [89].

### 4.4. Sulfide-Modified nZVI

Sulfide-modified nZVI is formed by combining Fe^0^ with elemental sulfur to create an iron sulfide protective shell on the nZVI surface [98]. This shell not only prevents self-corrosion and oxidation of nZVI but also alters its hydrophobicity, conductivity, and adsorption selectivity, facilitating electron transfer from the Fe^0^ core to the nZVI surface and improving the removal efficiency of recalcitrant pollutants. Li et al. [90] compared the performance of S-nZVI with nZVI in reducing TBBPA, finding that S-nZVI reduced over 90% of TBBPA within 24 h, achieving a reduction efficiency 1.65 times higher than that of nZVI. Additionally, sulfide modification enhanced the antioxidant properties of nZVI. Even after 11 weeks of aging, S-nZVI could still convert 56% of TBBPA within 24 h, whereas nZVI lost nearly 95% of its reduction capability after just 2 weeks. Cai et al. [91] optimized the S-nZVI/PS system to degrade bisphenol S (BPS) under optimal conditions, achieving maximum removal at an S/Fe molar ratio of 0.035. The BPS degradation rate was highly sensitive to PS concentration and S-nZVI dosage. Furthermore, the S-nZVI/PS@BC system exhibited superior atrazine (ATR) removal rates and stability due to the presence of ferric sulfide compounds compared to pure nZVI-PS and S-nZVI-PS. The experiments identified OH·, SO_4_^2−^, ^1^O_2_, and PFRs as the primary reactive oxygen species [92].

## 5. Recycling of Nano Zero-Valent Iron

The recycling and regeneration of nZVI are crucial for its reuse and economic feasibility in environmental remediation applications [99]. Effective regeneration not only restores the reactivity of nZVI but also ensures its sustained efficiency in activating persulfate for the degradation of environmental pollutants, including EDCs. Several methods have been developed to clean and regenerate spent nZVI, each with its advantages and limitations, as shown in Table 4.

The acid-washing method involves using acidic solutions, such as hydrochloric acid or sulfuric acid, to dissolve oxides and adsorbed pollutants on the nZVI surface [100]. This removes the passivation layer, restores the reactivity of zero-valent iron, and enhances its capacity to react with peroxynitrite, thereby improving its efficiency in oxidizing and degrading pollutants. Similarly, the alkaline washing method employs alkaline solutions, such as sodium hydroxide, to eliminate organic pollutants and oxidized layers on the nZVI surface, thereby maintaining its active sites and enhancing its activation efficiency against persulfate. This process also reduces issues related to agglomeration and passivation after use. The thermal treatment method uses high temperatures to remove organic matter and oxide layers from the nZVI surface while preventing further oxidation in an inert or reducing atmosphere [101]. This re-exposes the active surface of nZVI, enhancing its reactivity and ability to remove contaminants via persulfate activation. The chemical reduction method utilizes reducing agents like sodium borohydride to reduce the oxide layer of nZVI back to its zero-valent state. This effectively extends the service life of nZVI and enhances its ability to produce reducing intermediates during persulfate activation [102]. Ultrasonic treatment physically removes passivation layers and contaminants from the nZVI surface through ultrasonic vibrations, maintaining its active surface [103]. This method increases efficiency in the persulfate activation process and prevents particle aggregation and deactivation. Finally, electrochemical regeneration uses electrical currents to reduce oxides on the nZVI surface and simultaneously remove adsorbed contaminants [104]. This technique not only restores nZVI’s reactivity but also enhances its oxidative degradation capabilities during persulfate activation.

nZVI-activated persulfate technology is an efficient method suitable for in situ remediation, characterized by good environmental adaptability, low material production costs, and a reduced reliance on chemical agents, thereby lowering the risk of secondary pollution. The principles, advantages, disadvantages, and scope of application of the technologies that can be used are shown in Table 5.

These recycling and regeneration techniques are essential for prolonging the service life of nZVI and significantly improving its efficiency in the environmental remediation process. By maintaining the reactivity and stability of nZVI, these methods enable more sustainable and cost-effective treatment solutions for the degradation of EDCs and other persistent pollutants in water bodies.

## 6. Challenges and Recommendations for the Removal of EDCs by Nano Zero-Valent Iron-Activated Persulfate Systems

The primary advantage of nZVI as a persulfate catalyst is its ability to continuously provide ferrous ions. nZVI rapidly reduces persulfate to produce highly oxidizing sulfate radicals, which are capable of quickly degrading EDCs in water [105]. nZVI also exhibits high adsorption and reduction capacities, is easy to operate, and reacts under environmentally benign conditions without producing harmful by-products. Compared to traditional activation methods, the synergistic effect of nZVI and persulfate not only improves treatment efficiency but also reduces the amount of chemical reagents required, thereby lowering costs. However, several challenges persist in practical applications.

### 6.1. Stability and Persistence of nZVI

nZVI, as a highly reactive material, exhibits significant reactivity in water treatment processes due to its unique nanoscale size and high specific surface area. However, the stability and persistence of nZVI are major challenges in its practical applications. The small particle size and high surface activity of nZVI allows rapid release of Fe^2+^ to activate oxidants. However, as the reaction progresses, nZVI tends to agglomerate in aqueous solutions, forming larger aggregates that reduce its specific surface area and consequently decrease its contact efficiency with contaminants. Additionally, the surface of nZVI is prone to oxidation, forming passivation layers of iron oxides or hydroxides, which hinder electron transfer and significantly reduce the reductive capacity and reactivity of nZVI.

To address these issues, researchers have explored various strategies to enhance the stability and persistence of nZVI. For instance, surface modification with organic or inorganic stabilizers (such as chitosan, carbon materials, or clay minerals) can inhibit aggregation, and the synthesis of composite materials with magnetic or conductive properties can enhance reactivity. Moreover, these surface modifications can adjust the charge distribution and hydrophobicity of nZVI, optimizing its interaction with persulfate and improving reaction efficiency [106]. Despite these enhancements, maintaining high reactivity while improving stability remains a critical challenge in practical applications.

### 6.2. Optimization of Reaction Conditions

The performance of the nZVI–persulfate system is influenced by various reaction conditions, including pH, temperature, persulfate concentration, nZVI dosage, reaction time, and water quality characteristics (such as ionic composition and organic matter content). These factors significantly impact the reaction mechanisms and efficiency of the system. pH is one of the key factors controlling reaction activity; under low pH conditions, the presence of H⁺ helps maintain iron in a soluble state, thereby enhancing reaction rates. However, excessively low pH can lead to rapid dissolution and loss of iron, as noted in studies [107,108]. Conversely, exorbitant pH conditions can accelerate the passivation of nZVI surfaces, resulting in loss of activity [109]. Moreover, high temperatures may also promote the self-decomposition of persulfate, generating excessive reactive oxygen species that reduce reaction selectivity.

Optimizing these reaction conditions requires precise experimental design and data analysis while considering the diversity of water quality and operational simplicity in practical applications. Control of reaction time and temperature is particularly crucial for large-scale water treatment facilities, as these parameters directly affect operational costs and energy consumption. To achieve optimal pollutant removal efficiency, systematic optimization through response surface analysis, multi-factor optimization, and mathematical modeling is necessary to determine the best combination of operational parameters, balancing economic and environmental benefits [110].

### 6.3. Economic Feasibility

Economic feasibility is key to the large-scale application of the nZVI–persulfate system. Although this system demonstrates high pollutant removal efficiency under laboratory conditions, its high preparation and operational costs limit its broader application in engineering practices. The preparation of nZVI often involves complex chemical reduction processes with costly raw materials (such as sodium borohydride), and the management of by-products generated during production can be challenging. Moreover, the consumption of persulfate as an oxidant also presents economic constraints, with its cost and stability being critical considerations. Zeng et al. [111] utilized nZVI/BC to activate PS for the remediation of organic pollutant-contaminated soil at depths of 0–70 cm. After 360 days, the removal efficiencies for target pollutants, including 2-ethyl nitrobenzene (ENB, 1.47–1.56 mg/kg), biphenyl (BP, 0.19–0.21 mg/kg), 4-(methylsulfonyl) toluene (MST, 0.32–0.43 mg/kg), and phenylphenol (PP, 1.70–2.46 mg/kg), varied at different soil depths, achieving efficiencies of 99.7%, 99.1%, 99.9%, and 99.7%, respectively. Meanwhile, Song et al. [112] utilized micro/nanostructured zero-valent iron (nZVI), stearic acid-coated micro/nanostructured zero-valent iron (C-nZVI), and commercial micron-sized zero-valent iron (mZVI) to activate PS for the removal of PAHs from a contaminated site with an area of approximately 30 m^2^ and a depth of 4 m. After 104 days of treatment, the removal efficiencies of PAHs (~17 mg/kg) for nZVI-, C-nZVI-, and mZVI-activated PS were 82.21%, 62.78%, and 69.14%, respectively. Furthermore, the cost of nZVI prepared from ball-milled iron powder is only 8 to 13 USD/kg, while the price of mZVI ranges from 150 to 350 USD/kg.

To improve economic feasibility, researchers are developing more cost-effective nZVI synthesis methods, such as using industrial waste or natural minerals as iron sources or exploring biosynthesis and green reduction technologies [113]. Compared to traditional methods, these new approaches can significantly reduce production costs [114]. At the same time, an increasing number of studies are focusing on the preparation of composite materials, particularly the synthesis of carbon-based materials. Research indicates [115] that the cost of biochar is USD 272 per ton, while powdered activated carbon costs USD 750 per ton and granular activated carbon costs as much as USD 2000 per ton. In contrast, the average cost of wood biochar is only USD 24.8 per ton. This indicates that future development should increasingly favor the use of green raw materials for biochar production. Additionally, strategies such as reducing reagent usage, optimizing reaction conditions to enhance persulfate utilization, and developing techniques for the recovery and reuse of by-products can effectively lower overall costs. Furthermore, life cycle analysis and cost–benefit assessments of the system can help to understand the economic advantages and limitations of this technology across various application scenarios [99,116].

### 6.4. Environmental Risks and Mitigation

The nZVI–persulfate system has clear advantages in removing EDCs and other organic pollutants, so its potential environmental impact cannot be overlooked. The use of nZVI may lead to the accumulation of iron ions and their oxidation products in water bodies or soils, which could disturb ecosystem balance. Research indicates that the aggregation and diffusion of nZVI can pose toxicity risks to aquatic organisms, primarily through mechanisms such as oxidative stress and interference with cellular functions [117,118]. Additionally, while the activation of persulfate generates various ROS, including hydroxyl and sulfate radicals that are effective in degrading pollutants, these species may also adversely affect non-target organisms due to their strong oxidative properties [59].

To mitigate environmental risks, researchers have proposed several green improvement measures, such as optimizing the dosage of nZVI and reaction conditions to minimize secondary pollution and developing biodegradable stabilizers or encapsulation materials to control the release of nZVI [119]. Meanwhile, it is also crucial to ensure that the concentration of iron after treatment does not exceed legal limits, as exceeding these limits incurs additional costs for iron removal. Furthermore, it is essential to evaluate the toxicity and biodegradability of reaction by-products to ensure that they do not pose long-term risks to the environment and human health [120]. Comprehensive consideration of environmental impacts, along with the establishment of scientific usage guidelines and safety management measures, is crucial to ensure the safe and effective application of the nZVI–persulfate system in real-world scenarios.

## 7. Conclusions

nZVI emerges as a highly effective catalyst for persulfate activation, enabling the efficient degradation of EDCs. Modifications to nZVI enhance its reactivity and stability, addressing key challenges in environmental remediation. However, issues such as particle agglomeration, oxidation, and economic feasibility must be resolved for broader applications. Future research should focus on developing sustainable, cost-effective modifications and integrating nZVI with complementary technologies. This approach could establish nZVI as a critical tool in advanced water treatment, significantly advancing environmental sustainability efforts.

## Figures and Tables

**Table 1:** The mechanisms, advantages, and disadvantages of commonly used EDC removal methods.

Method Categories	Mechanism of Action	Method Advantages	Method Deficiency
Adsorption method	Utilizes physical or chemical interaction between the adsorbent surface and the EDC molecules	Simple operation, low equipment requirements, can be applied to different water quality	Adsorbents need to be replaced or regenerated periodically and their limited adsorption capacity requires regular monitoring
Membrane separation method	The EDCs are separated from water by the physical sieving action of the membrane	Can effectively remove small-molecule EDCs without generating secondary pollution	Membrane fouling and failure necessitate regular cleaning or replacement and high initial investment and maintenance costs
Biodegradation method	Microorganisms or plants metabolize EDCs, converting them into harmless substances	Environmentally friendly, can occur naturally	Treatment rates are slower, and efficiency is influenced by environmental factors, requires specific microbial or plant communities
Chemical precipitation method	Formation of insoluble precipitates from EDCs by addition of precipitants for easy removal	Simple operation with clear treatment effects	Effective and less versatile only for specific EDCs and may generate solid waste, requiring disposal
Photocatalytic method	Utilize photocatalysts to generate electrons and holes upon light irradiation, thereby degrading EDCs	Can be carried out at room temperature and pressure, easy to operate, high degradation efficiency, wide range of applications	Dependent on light source, less effective in dark environments

**Table 2:** The standard oxidation electrode potential of common active species.

Number	Oxidizing Agent	Standard Oxidation Electrode Potential (*E*^0^/V)
1	OH	2.8
2	SO_4_^−^	2.5–3.1
3	HO_2_	1.65
4	O_3_	2.08
5	H_2_O_2_	1.76
6	S_2_O_8_^2−^	2.01

**Table 3:** Comparison of pollutant degradation by nZVI–persulfate system with different modification methods.

Contaminant	Concentration of Pollutant	Modifying Methods	Degradation Rate	Modified nZVI Dosage	PS Dosage	Reaction Condition	Dominant Free Radical	Reaction Product	Reference
TC	50–300 mg/L	PVP-nZVI	100%	0.1 g/L		pH = 6.5, 25 °C, 150 rpm	OH	C_20_H_23_NO_7_, C_20_H_26_O_4_, C_19_H_26_O	[81]
PAHs	100 mg/kg	SiO_2_/nZVI	75%–87%	2 g/L	10 mL	25 ± 1 °C	SO_4_^−^		[82]
2,4-DCP	40 mg kg^−1^	BC-nZVI	91%	3.0 g/L	6 mM	pH = 4.51, 20 °C, 240 min, 200 rpm	SO_4_^−^	2-chlorohydroxyaniline, 3,5-dichlorocatechol, phenol, transbutenoic acid and acetic acid	[83]
BDE209	10 ± 0.5 mg·kg^−1^	BC-nZVI	82.06%	Vary with mole ratio	0.1 M	pH = 3, 40 °C, 240 min	SO_4_^−^, OH	Low brominated diphenyl ethers, phenol substances, CO_2_, H_2_O, Br- and short-chain acids	[84]
BPA	10 mg/L	BC-nZVI	98%	1.0 g/L	0.75 mM	pH = 3.0 ± 0.03, 25 °C, 150 rpm	SO_4_^−^, OH	p-Isopropylphenol, 4-Isopropylphenol, 4-Hydroxyacetylbenzene, hydroquinone, transbutenoic acid and 2-hydroxypropionic acid	[85]
ATZ	23.6 mg/kg	BC-nZVI	96%	2.0 g/L	4.0 mM	pH = 6.93, 240 min, 150 rpm	SO_4_^−^, OH, ^1^O_2_	De-alkylation, dechlorination, and hydroxylation products	[86]
BDE209	2 mg/L	Fe/Ni	≈100%	4 g/L		pH = 6.09, 28 ± 2 °C, 200 rpm		BDE206, BDE207	[87]
DCP		Fe/Cu	86%	0.63 g/L	6 mmol/L	pH = 3, 25 °C	SO_4_·^−^, OH	2-Chlorohydroxybenzoquinone, 2-chlorobenzoquinone, oxalate ion	[88]
DCP	15 mg/L	Fe/Cu	≈100%	0.1 g/L	2 mM	pH ~3.3, 25 °C, 150rpm	SO_4_·^−^, OH	2-Chlorohydroxybenzoquinone, 2-chlorobenzoquinone, oxalate ion	[89]
TBBPA	20 mg/L	S-nZVI	≥90%	2.3 g/L		Dark, 25 °C, 300 rpm		BPA, tri-BBPA, di-BBPA, mono-BBPA	[90]
BPS	20 μM	S-nZVI	97.7%	30 mg/L	1 mM	pH = 5.6 ± 0.2, 25 °C	SO_4_·^−^, OH	BPA, tri-BBPA, di-BBPA, mono-BBPA	[91]
ATR	10 mg/L	S-nZVI@BC	100%	0.1 g/L	1 mM	pH 2.86–10.53, 25 °C	SO_4_^−^, OH, ^1^O_2_	2-hydroxyatrazine, 2,4-dichlorophenol, 4-amino-2-chlorotriazine, 2-amino-4-chlorotriazine	[92]

**Table 4:** Comparison of the principles, advantages and disadvantages, and scope of application of nZVI regeneration methods.

Method	Theory	Advantage	Defect	Range of Application
Acid and Alkaline Washing	Clean the nZVI surface using an acidic or alkaline solution to remove the passivation layer	Effective restoration of surface activity and relatively simple to implement	The complexity of waste liquid treatment may lead to loss of nZVI	Widely used for regeneration of nZVI in the field of water treatment
Thermal Treatment	Removal of impurities and passivation layers from the nZVI surface by heating	Complete removal of organic and inorganic substances with high recovery rates	May lead to nZVI agglomeration or morphological changes and high energy consumption	Suitable for removing organic/inorganic impurities from various environments
Chemical Reduction	Oxidized nZVI was restored to a zero-valent state using a reducing agent	Significant restoration of reactivity, allowing for directional operation	Introduction of new chemicals, complex process	Suitable for applications requiring specific restoration of reactivity
Ultrasonic Treatment	Removal of contaminants and dispersion of agglomerated nZVI particles using high-frequency ultrasound	High cleaning efficiency and effective dispersion of particles	High cost of equipment, ultrasonic intensity and time need to be precisely controlled	Suitable for scenarios where particle dispersion is critical
Electrochemical Regeneration	Restore the active state of nZVI by electrochemical reaction	High recovery efficiency without the need for additional chemical reagents	Requires complex equipment and high energy consumption	Effective in restoring nZVI reactivity without the addition of external chemicals

**Table 5:** Comparison of available techniques for in situ remediation by nZVI-activated persulfate technology.

Method	Theory	Advantage	Defect	Range of Application
In situ chemical oxidation method	Activation of persulfate using nZVI generates strong oxidizing radicals that oxidize and degrade pollutants in soil and water	Capable of degrading a wide range of pollutants, fast reaction rate, wide applicability	Reaction conditions are demanding and require controlled reaction environments	Groundwater pollution control, contaminated soil remediation, industrial wastewater treatment
Injection method	After mixing nZVI with persulfate, the activator is introduced directly into the contaminated area by injection techniques to create an oxidizing environment	Ability to deliver remediation chemicals directly to the source of contamination, suitable for rapid remediation emergencies	Requires specific equipment for injection, limited effectiveness in confined or hard-to-reach areas	Small-scale contaminated site remediation, water pollution management, areas with high groundwater table
Solid phase reaction method	The solid form of nZVI combined with persulfate is applied to contaminated soils or sediments and activated by a solid-phase reaction	Easy to store and transport for long-term contaminant removal	Solid-phase reactions may have low reaction rates and exposure to contaminants may not be uniform	Solid waste management, soil contamination remediation, sediment treatment
Microbial assisted remediation method	Combining nZVI and persulfate technologies with bioremediation to utilize microbial metabolic processes to further degrade contaminants	The synergistic effect of microbial and chemical oxidation improves overall remediation efficiency and microbial degradation reduces the risk of secondary contamination	Microbial activity and growth are affected by environmental conditions and bioremediation takes longer to take effect	Treatment of organic pollutants, long-term remediation of contaminated soils, remediation of water bodies with suitable environmental conditions

## Data Availability

Data sharing is not applicable to this article. All data are contained within this manuscript.
